# Chunking in working memory via content-free labels

**DOI:** 10.1038/s41598-017-18157-5

**Published:** 2018-01-08

**Authors:** Liqiang Huang, Edward Awh

**Affiliations:** 1 Department of Psychology, The Chinese University of Hong Kong, ShaTin, Hong Kong; 2 Department of Psychology, University of Chicago, Chicago Illinois, USA

## Abstract

A recent study found that visual working memory performance was enhanced when pairs of colors were predictably paired, and it was interpreted as a form of “memory compression” which implies that more colors could be stored online in a more efficient format. Here we propose an alternative hypothesis that does not entail any increase in the number of individuated representations stored online. Instead, familiar ensembles of items may be attached to a content-free label (e.g., remembering red-white-blue as “American flag”) that can be used to retrieve the constituents of a chunk when they are needed to guide a response. If accessing “compressed” memories requires an additional retrieval process, then access to compressed items should be slower than for uncompressed items. Indeed, Experiments 1 (visual) and 2 (verbal) showed that response times were substantially longer in patterned (i.e., compressed) than in control conditions. In Experiments 3 and 4, regularity-based advantages were eliminated with brief (1000 or 875 ms) response deadlines, in line with our hypothesis that accessing compressed memories requires a slow retrieval process. In sum, while statistical regularities can enable access to larger amounts of information, this information may not be available “online” in the same way as singleton items.

## Introduction

Working memory (WM) has been conceptualized as an “online” storage system that enables the active maintenance of information in mind. A large body of research has examined the nature of capacity limits in visual WM, and one clear consensus is that there are sharp limits in visual WM capacity^1–7^. Various studies, however, have shown that performance in visual WM tasks can be enhanced when there are predictable associations between the items to be stored^1,8^. For example, Brady *et al*. showed that performance in a change detection task could be dramatically improved when specific colors in the sample display were presented in predictable pairs^1^ (e.g., a yellow disk surrounded by a red ring, or a yellow disc always adjacent to a red disc). Indeed, this study demonstrated that observers were virtually optimal in exploiting these regularities. Taking an information theory perspective^9^, Brady and colleagues referred to this regularity-based advantage as “memory compression”.

The concept of “memory compression” implies that visual WM capacity had been changed so that a larger number of individual items were stored online. By contrast, another possibility is that memory compression may reflect the operation of a traditional chunking process in which the observers have learned to associate a set of colors, and then retrieve the associated colors only when they are needed to guide behavior. This kind of learning is common. For example, observers can remember six colors black-yellow-red-green-white-orange as “Belgium and Ireland”, and report these six colors perfectly in a subsequent test, but this does not mean their visual WM capacity has increased to include six individual colors. Similarly, perhaps in Brady *et al*.^1^ the observers have explicitly learned the regular chunks (i.e., color pairs) and used them to perform the task without concurrently storing all of the relevant color in WM.

**Figure 1:**
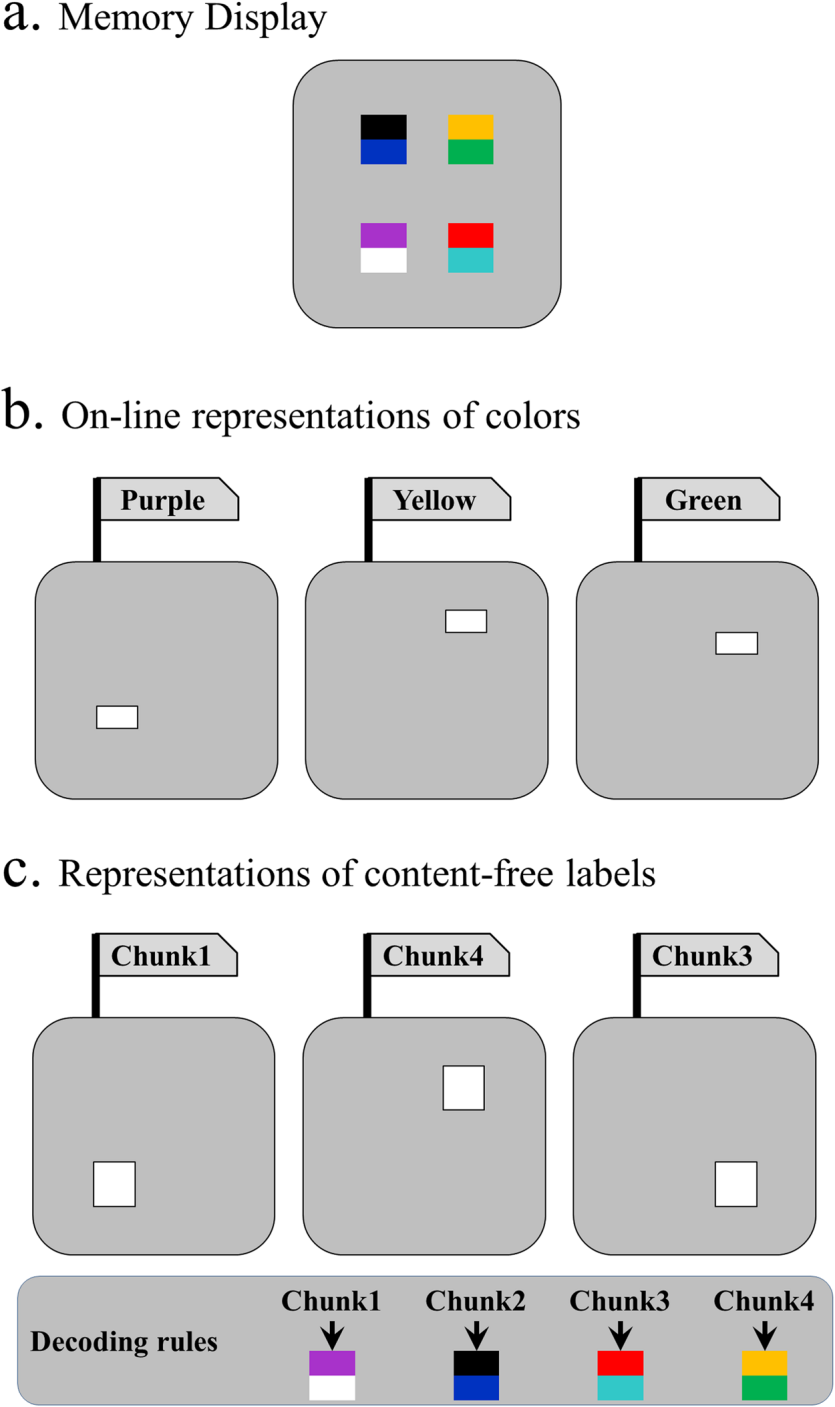
On-line and chunked representations of colors. For the same set of stimuli display (panel a), the observers may try to memorize it directly with on-line representations (panel b), or by referring to offline representations (panel c). In the latter, content-free labels of chunks replace the raw color information, and the “decoding rules” of labels have to be stored as offline representations.

Figure 1 illustrates how a “memory compression” could be implemented by a chunking process. Consider an observer with a memory capacity of three chunks. For the sample display illustrated in Fig. 1a, this observer could hold three individual colors in an online state in visual WM (Fig. 1b), or if an observer has learned the color pairs as chunks, then he or she could retain content-free abstract labels for these chunks in visual WM while the specific content of the items within each pair was stored separately (Fig. 1c).

## Chunking

Traditionally, chunking refers to the process of giving a label to a set of information so this set can be efficiently represented and used as an integrated unit. For an analogy, when two commercial plans are being considered, they may be simply referred to as Plan A and B so that their details can be omitted in the discussion. When necessary, these details can be retrieved from the relevant documents. Chunking has been an important topic of cognitive psychology since the early pioneers^10–12^. In the classic work of Miller^12^, he proposed that the human cognitive capacity is limited to several chunks. Critically, even if the number of chunks remains constant, it is possible for observers to increase the amount of information referenced by a chunk via associative learning. For example, classic studies of chess expertise^10,11,13^ showed that players at and above master level remember the positions in real games much better than novices. However, their memory of random positions is basically the same as that of the novices. Thus, the chess masters do not have a greater number of chunks than novices in their memory. Instead, they know how to represent multiple pieces within common game patterns as chunks, whereas the novices do not.

## Content-Free Label in Chunking

There are several reasons why we believe that chunking may be based on content-free labels. First, from a computational point of view, the use of content-free label seems like an obvious strategy. In a computer system, a handle is an abstract reference to a resource, and the handle is content-free. A computational system that is not designed in this way would be inefficient when the label alone is sufficient to retrieve each element when necessary. For example, a file name like “cities.txt” could be used for a file that includes the names of five cities (Rome, Paris, Boston, Tokyo, Beijing) instead of a file name like “RomeParisBostonTokyoBeijing.txt”. Clearly, the former strategy is more efficient when the individual city names are not yet needed. Similarly, content-free labels could provide an efficient way to handle familiar ensembles of information in a WM task, especially when there is motivation to simultaneously store information about other stimuli.

Second, the use of content-free labels in chunking seems obvious in some cases. For example, someone remembering the string “internationalizationcongratulationmisinterpretation” would probably agree that they are not holding online individuated representations for each letter in this string, even if every letter could be recalled perfectly given sufficient time. Similarly, a Chinese reader remembering the visual pattern  would probably agree that they do not maintain online individuated representations for all the visual details (e.g., orientations and sizes of each part of a stroke), even if those details can be recalled perfectly given enough time. A natural explanation for this introspection is that people hold content-free labels in mind rather than the individual items that comprise each ensemble.

Third, the use of content-free labels seems to be naturally implied by some other important concepts such as type/token distinction^14^ or object files^15^. In the type/token distinction, if a type repeats frequently as many tokens, then it seems plausible that each of the token will only represent a “content-free label” and ignore the visual details (i.e., leave them in LTM as we assumed). For example, when there are many identical cars in a visual scene, it seems plausible that the cognitive system will represent each token of car as a “content-free label”. This type/token distinction has been applied to explain various phenomena and/or mechanisms such as attentional blink^16^ and visual WM^17^. In all these cases, it seems natural that the tokens function as content-free labels.

## Cost of Decoding

Although the introspection is obvious in the cases described above, it is not so obvious in borderline cases. For example, it is not obvious whether the memory of a “Belgium flag” contains the active representation of the three colors or not, or the memory of a word “dog” contains active representations of the three letters or not. Nevertheless, our hypothesis is that even in such borderline cases, chunks are maintained via content-free labels that do not give access to the individual elements in the chunk. Instead, an individual element within a chunk can only be used after a *decoding* process that enables the elements of a chunk to be *actively represented as individuated items in WM*. We reasoned that this retrieval process should take time, thereby delaying responses that are guided by those individuated representations. Therefore, we predict that *access to information, and consequently response times in a visual WM task, will be slower when subjects are exploiting statistical regularities than when they are not*. The present experiments tested this prediction.

## Chunking in Verbal WM

Although memory compression is a relatively new notion in visual WM, the role of chunking has been well-established in verbal WM^18^. For example, one can more easily remember the string “fbicbsibmirs” than a randomly-generated string of the same length. We propose that the “content-free labels which refers to offline representations” are general to all types of chunking and are therefore also responsible for chunking in verbal WM. In the “fbicbsibmirs” example, one will only remember four acronyms, FBI, CBS, IBM, and IRS, as content-free labels, but the composition of each acronym is not represented on-line and is retrieved from an offline state when it is needed. Our hypothesis falls in line with the interpretation offered by Chen and Cowan^8^ when they trained observers to remember pairs of familiar words, and found that subjects could maintain an equivalent number of these pairs as they could word singletons. While their study was focused on a “core capacity” that could be defined by the number of chunks stored, our study focuses on the predicted cost of unpacking those chunks when a decision requires access to the constituents of a chunk. Thus, we predict that in a task which requires information about an individual letter (e.g., cuing a location in a sequence and report the letter), responses will be slower for a “chunked string” than a string consisting of random letters.

## Experiment 1–2: Slower Response for Patterned Blocks

Experiments 1-2 tested whether access time for items in patterned blocks (i.e., blocks with statistical regularities) would be slower than for items in control blocks (i.e., blocks without statistical regularities), in line with the above-mentioned hypothesis that improved memory performance in the patterned blocks was accompanied by a cost of decoding. Experiment 1 used colors as stimuli to test visual WM, whereas Experiment 2 used letters as stimuli to test verbal WM.

### Method

In all experiments, the stimuli were presented on a 1,024 × 768 pixels CRT color monitor. The observers viewed the display from a distance of about 60 cm and entered responses using a keyboard. The program was written in Microsoft Visual Basic 6.0 and was run on Microsoft Windows XP using timing routines tested with the Blackbox Toolkit (Blackbox Toolkit Ltd., York, England).

#### Participants

Students at the Chinese University of Hong Kong completed Experiments 1 and 2 for a compensation of HK$50. There were 32 participants in each of Experiments 1 and 2. All had normal or corrected-to-normal vision.

All experiments of the present study were carried out in accordance with approved guidelines. The consent form and experimental procedures received prior ethical approval from research ethics committee of the Chinese University of Hong Kong. Informed consent was obtained from each participant.

#### Stimuli

**Figure 2:**
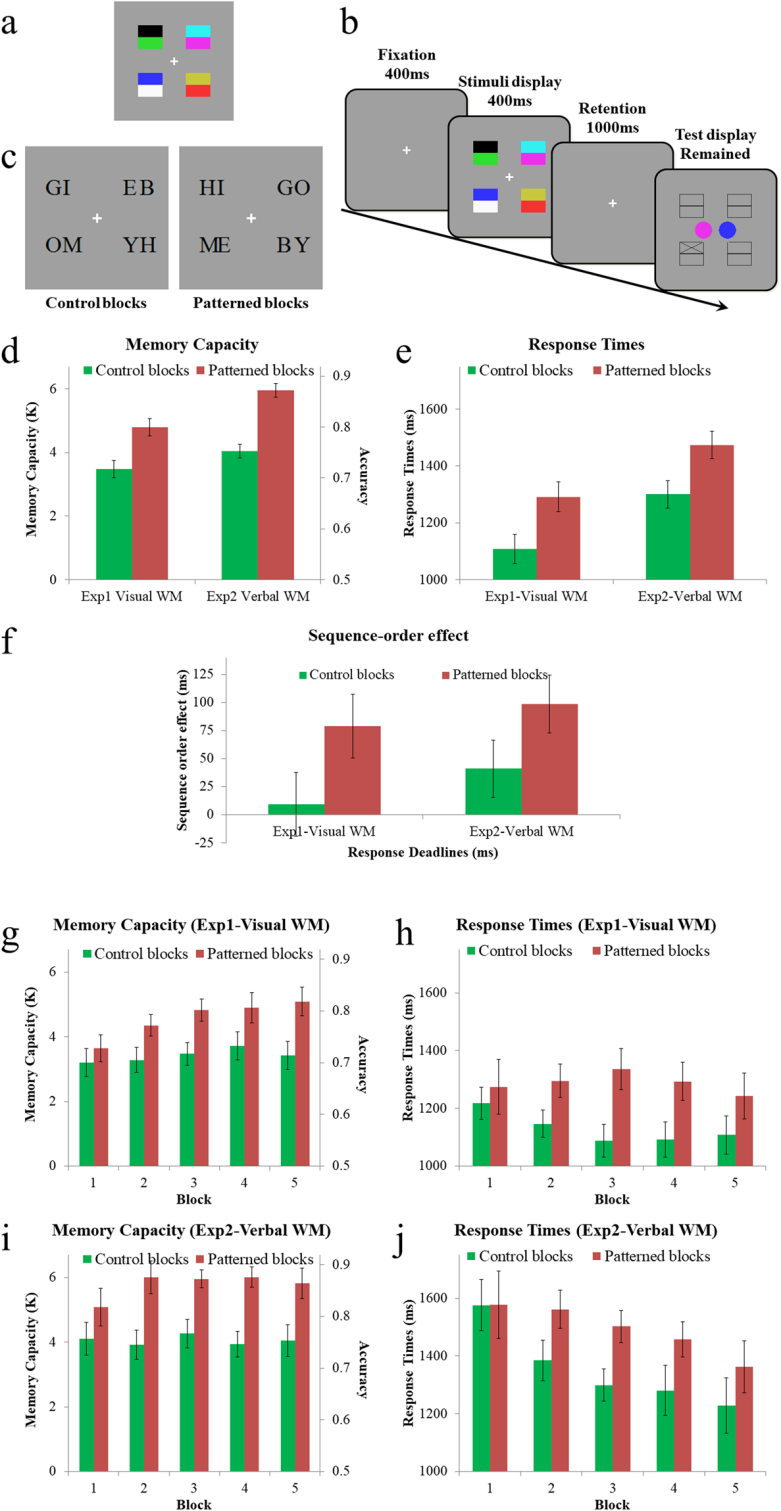
Methods and results of Experiment 1–2. Panel (a and c) respectively shows sample stimuli displays of 8 colors (Experiment 1) and 8 letters (Experiment 2). Panel (b) shows the sequence of presentations: the stimuli were presented for 400 ms and then disappeared. One second following its disappearance, the test display was presented marking 1 of the 8 positions and showing two choices, one of which had appeared in this marked position. Panel d shows the memory capacities (left-side axis), which was estimated from the accuracies of responses (right-side axis): K = (accuracy-0.5) × 16. Memory capacity significantly increased in patterned blocks than control blocks, confirming the previous finding. More importantly, the response times were significantly slower in the patterned than the control blocks (Panel e). Panel f shows the sequence-order effect: the RT advantage of top-color over bottom-color in Experiment 1, or left-side letter over right-side letter in Experiment 2. The sequence-order effect was significantly smaller in control blocks than in patterned blocks, and suggests that when a pair in the patterned blocks needs to be decoded, the individual items are retrieved in a stereotyped order. Panels (g–j) show the time-course of these effects. Consistent with the Brady, *et al*.^1^, for both accuracy (i.e., memory capacity) and RTs, the differences between the patterned and control blocks were relatively small in Block 1, and these differences gradually increase with more learning processing. Error bars show within-subject 95% confidence intervals^32^.

Sample stimuli displays of Experiments 1 and 2 are respectively shown in Fig. 2a and c. In Experiment 1, eight colors were presented, two on each of the 4 corners (i.e., left-top, left-bottom, right-top, right-bottom), against a gray background (Fig. 2a). The two colors of each corner occupy the top and bottom half of a 1.04 cm × 1.04 cm square, which is 1.47 cm away from the center of the display. For each observer, the 8 colors (red, green, yellow, blue, cyan, purple, black, and white) were divided into 4 pairs. This division was randomized across different observers (i.e., partial counterbalancing). In each trial of patterned blocks, the 4 pairs were randomly arranged in the 4 corners. In other words, the color values in each pair (e.g., red-top-black-bottom) were constant, but that the position of each pair was randomized. In each trial of control blocks, the 8 colors were randomly arranged in the 8 possible positions.

In Experiment 2, the stimuli consisted of 8 black letters (Fig. 1c). The two letters of each corner occupied the left and right half. The 8 letters were divided into 4 pairs (GO, HI, BY, ME), which were all very common words. In each trial of patterned blocks, the 4 words were randomly arranged in the 4 corners. In each trial of control blocks, the 8 letters were randomly arranged in the 8 possible positions.

#### Procedure

The sequence of presentations is shown in Fig. 2b. At the start of each trial, a small white fixation cross was presented in the center of the display for 400 ms, and then the stimuli were presented for 400 ms and then disappeared. One second following the disappearance of the stimuli, the test display was presented marking 1 of the 8 positions and showing two choices (e.g., 2 colors in Experiments 1 and 2 letters in Experiment 2), one of which had appeared in this marked position and the other (i.e., the incorrect choice) was randomly selected from the other seven possible items. This test display remained on the display until the observers responded. The observers had to decide which of two items in the test display had been presented on the marked position of the stimuli display and then press one of two adjacent keys (“j” for the left item and “k” for the right item) to indicate their response. They were asked to give first priority to the accuracy of their responses and also try to respond as quickly as possible. After responding, the observers heard either a pleasant or an unpleasant tone to indicate whether their response was correct, and the next trial began 400 ms later. Each observer completed 10 blocks (60 trials per block). For half of the participants, blocks 1–5 were patterned blocks whereas blocks 6–10 were control blocks, whereas for the other half, this order was reversed. The first patterned block and the first control block (i.e., blocks 1 & 6) were regarded as learning of the presence/absence of color patterns and excluded from the analysis.

### Results and discussion

As shown in Fig. 2d, the memory capacity, as estimated with Cowan’s k^18,19^, significantly increased in patterned blocks relative to control blocks in both experiments (Experiment 1: 4.79 vs. 3.48, t (31) = 5.04, Cohen’s d = 0.89, p < 0.0001; Experiments 2: 5.95 vs. 4.04, t (31) = 9.15, Cohen’s d = 1.62, p < 0.0001). These results confirm the finding of Brady, *et al*.^1^ as well as the classic chunking in verbal WM.

In Experiments 1–2, response times were calculated over correct trials only. The RT outliers of each participant were excluded by first removing all response time values greater than 10,000 ms, and then removing all values beyond 3SDs. As shown in Fig. 2e, the response times were substantially slower in the patterned than the control blocks (Experiments 1: 1291 ms vs. 1108 ms, t (31) = 3.61, Cohen’s d = 0.64, p < 0.0005; Experiments 2: 1474 ms vs. 1300 ms, t (31) = 3.67, Cohen’s d = 0.65, p < 0.0005), confirming the cost of decoding in both visual and verbal WM.

A further analysis revealed an interesting sequence-order effect on the response times: for each pair in the patterned blocks, the response to the top-color was significantly faster than that to the bottom-color in Experiments 1 (1256 ms vs. 1335 ms, t (31) = 2.76, p < 0.005), and the response to the left-side letter was significantly faster than that to the right-side letter in Experiments 2 (1425 ms vs. 1524 ms, t (31) = 3.92, p < 0.0005). As shown in Fig. 2f, this sequence-order effect was significantly smaller in control blocks than in patterned blocks (Experiments 1: 9 ms vs. 79 ms, t (31) = 2.51, p < 0.01; Experiments 2: 41 ms vs. 99 ms, t (31) = 2.29, p < 0.02). These results suggest that when a pair needs to be decoded, the individual items are retrieved in a stereotyped order, from top-color to bottom-color, or from left-side letter to right-side letter.

The time-course of these effects are illustrated in Fig. 2g–j. The block number refers to the order of a block in its own type. For example, block 2 of patterned blocks mean the second block if an observer run in the order of patterned-control, but the seventh block if an observer run in the order of control-patterned. Consistent with the Brady, *et al*.^1^, for both accuracy (i.e., memory capacity) and RTs, the differences between the patterned and control blocks were relatively small in Block 1, and these differences gradually increase with more learning.

## Experiments 3–4: A Response Deadline Method

Experiments 1–2 suggest that access times for compressed items may be slower than for uncompressed items, in line with a chunking account in which the details about items within an ensemble must be retrieved before they can be used to guide behavior. Experiments 3–4 provided converging evidence for this hypothesis using a response-deadline approach in which observers were forced to respond within a specified amount of time. If the additional information available in the patterned condition has to be retrieved from an offline state, then the regularity-based advantage should be abolished when brief response deadlines preclude successful retrieval. By contrast, if memory compression enables a larger number of items to be stored online in WM, then a regularity-based advantage should be evident even for brief response deadlines. Thus, Experiments 3–4 enabled a clearer measure of the temporal dynamics of information access in the patterned and control blocks.

### Method

The method of Experiments 3–4 was identical to that of Experiments 1–2 with the following exceptions. There were 40 observers in each experiment. The two items (i.e., two choices) in the test display shrank from view at different rates, and the observers were instructed to respond before the disappearance of these items. If the observers failed to respond before their disappearance, then this trial was marked as an incorrect response with the feedback of an unpleasant sound. The duration of test-displays could be either brief (1000 ms in Experiment 3; 875 ms in Experiment 4) or prolonged (2500 ms), and the two levels of durations (prolonged vs. brief) were randomly intermixed within each block.

## Results and Discussion

**Figure 3:**
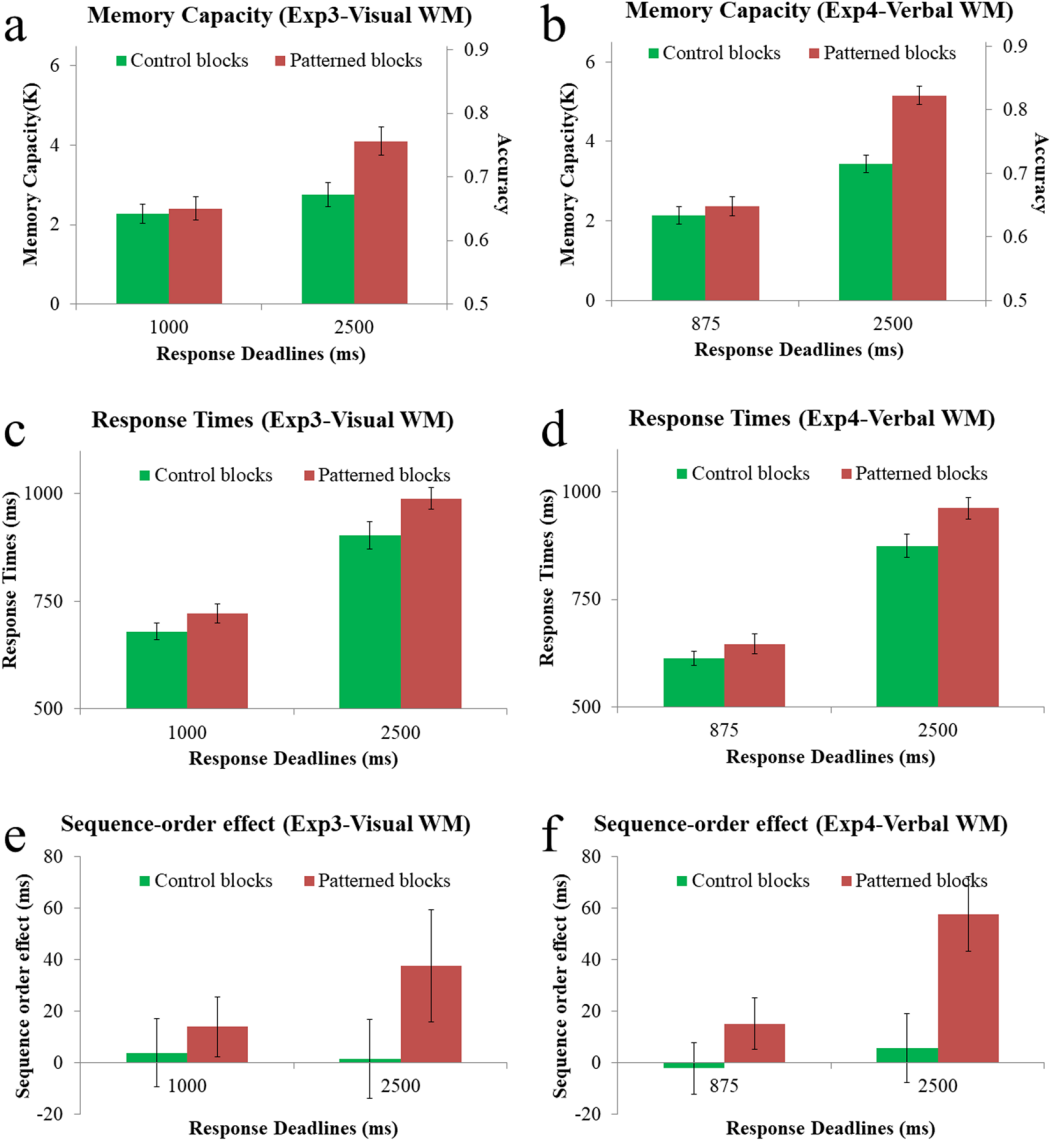
Results of Experiments 3–4. Panel (a,c) shows the memory capacities (left-side axis), which was estimated from the accuracies of responses (right-side axis): K = (accuracy-0.5) × 16. For memory capacities, there is a clear interaction as predicted by the chunking account: There is a substantial advantage in the patterned condition compared to the control condition in the prolonged response deadline (2500 ms), but this advantage almost disappeared in the brief response deadline (1000 ms & 875 ms in Experiments 3 and 4). The response times data (panel b,d) confirms the finding of Experiments 1–2. The response times were significantly slower in the patterned than the control blocks in all levels. We also analyzed the sequence-order effects (panel e,f). There were significant sequence-order effects in patterned condition in the prolonged response, and these sequence-order effects were reduced in the brief response deadlines. Error bars show within-subject 95% confidence intervals^32^.

The results of Experiments 3 & 4 are shown in Fig. 3. There were clear interactions between the effects of regularities and the time available for responding (Fig. 3a and b). In the prolonged response deadline, a substantial advantage was observed in the patterned condition compared to the control condition (Experiment 3: 4.10 vs. 2.76; Experiment 4: 5.16 vs. 3.43). However, this advantage almost disappeared in the brief response deadline (Experiment 3: 2.41 vs. 2.28; Experiment 4: 2.37 vs. 2.14). The interactions between response deadline (prolonged vs. brief) and regularities (patterned vs. control) were significant in both Experiment 3 (f (1, 39) = 28.40, η_p_^2^ = 0.42, p < 0.0001) and Experiment 4 (f (1, 39) = 28.97, η_p_^2^ = 0.43, p < 0.0001). Specifically, these interactions means that there were reliable increases in the size of the regularity-based advantage at prolonged versus brief response deadlines (Experiment 3: 1.35 vs. 0.13; Experiment 4: 1.73 vs. 0.23).

To describe these interactions from another perspective, there are modest improvements of performance over time in control blocks. This is natural because the RT-accuracy trade-off is a ubiquitous phenomenon and there are various factors that impair the performance when time is limited. More critically, the improvements of performance over time are much greater in patterned than in control blocks (1.69 vs. 0.48 in Experiment 3; 2.79 vs. 1.29 in Experiment 4). This implies that the time is much more critical in patterned than the control blocks, presumably because exploiting the regularities in the patterned blocks requires time for the retrieval of offline representations.

We did not attempt to exclude RT outliers in Experiments 3–4 because the RTs were already limited by the response deadlines. The response time data of the correct trials (Fig. 3c,d) confirmed the finding of Experiment 1. The response times were significantly slower in the patterned than the control blocks in all levels of response deadlines (p < 0.001 for all levels).

We also analyzed the sequence-order effects in the same way as in Experiments 1–2. As shown in Fig. 3e,f, the sequence-order effects were generally smaller in Experiments 3–4 than in Experiments 1–2. This is probably because these effects were weakened by the time-pressure of the response deadline. Importantly, the pattern of results was fairly consistent with our proposal for content-free labels. There were significant sequence-order effects in patterned condition in the prolonged response deadline in both Experiment 3 (f (1, 39) = 5.81, η_p_^2^ = 0.13, p < 0.025) and Experiment 4 (f (1, 39) = 33.81, η_p_^2^ = 0.46, p < 0.0001). These sequence-order effects were reduced in the brief response deadlines in both Experiment 3 (f (1, 39) = 2.90, η_p_^2^ = 0.07, p < 0.1) and Experiment 4 (f (1, 39) = 16.11, η_p_^2^ = 0.29, p < 0.0005). Moreover, the interaction between response deadline (prolonged vs. brief) and condition (patterned vs. control) were nearly significant in Experiment 3 (f (1, 39) = 3.36, η_p_^2^ = 0.08, p < 0.08) and significant in Experiment 4 (f (1, 39) = 8.20, η_p_^2^ = 0.17, p < 0.01).

To sum up, the results of Experiments 3–4 again clearly show that although additional information is available in the patterned trials, accessing that additional information takes a substantial amount of time. At brief response deadlines, there was little trace of an advantage in the patterned condition, inconsistent with the claim that subjects had a larger number of items represented online in the patterned condition. Instead, this empirical pattern is consistent with our hypothesis that memory compression is based on a slow process for retrieving information about the constituents of a chunk.

## General Discussion

To summarize our findings, Experiments 1 and 2 replicated previous demonstrations that statistical regularities enable access to a larger number of feature values in a visual WM task^1^ and in a verbal WM task^18^. This advantage, however, was accompanied by a marked slowing in the speed with which those compressed representations could be used to guide behavior. Experiments 3–4 provided converging evidence for this conclusion using a response deadline procedure. With brief response deadlines, performance was equivalent in the patterned and control conditions, in line with our hypothesis that accessing “compressed” information from regular pairs requires a relatively slow retrieval process that could not be completed in the brief deadline conditions. Thus, we propose that regularity-based advantages in both visual WM (i.e., memory compression effects) and verbal WM (i.e., the chunking advantage) may reflect a dynamic collaboration between online and offline representations, such that content-free labels of chunks are stored online while the values of the associated items are retrieved from an offline state when they are needed to guide behavior. Critically, this suggests that regularity-based advantages may not change the number of individuated representations that can be stored in WM, in line with Chen and Cowan’s observation that subjects could store the same number of well-learned word pairs as they did word singletons. The present work extends this finding by demonstrating the temporal cost of decoding the contents of a chunk.

### Relation to Models of WM

In the traditional framework of multi-store models^20,21^ which holds that the underlying mechanisms of WM and long-term memory (LTM) are separate from each other, the notion of content-free labels implies that only those labels are represented online in WM, while the actual content of the chunks are stored offline in LTM. Thus, information about individual elements within a chunk is not available until it is retrieved into WM from LTM. That said, the notion of content-free labels is equally compatible with unitary-store models^18,22–25^ that posit a common representational space for WM and LTM, such that WM is an activated subset of LTM. Here, the notion of content-free label implies that the labels – which are devoid of individual item information – are held in an activated state, while the details about the items associated with those labels must first be moved into an active state if they are to guide behavior. Thus, activation of a content-free label does not give access to an individual element within the associated chunk until time is taken to shift that information into an active state. In both cases above, the critical point of the notion of content-free label is that an individual element within a chunk can only be used after a *decoding* process.

### Storage of the Content of Chunks

We assume that the contents of chunks are stored in LTM because it seems both burdensome and unnecessary to keep them in WM. Nevertheless, the prediction on the cost of decoding does not depend on this assumption of the locus of the storage of the content of chunks. The critical prediction of the present study, namely the cost of decoding, relies on the assumption that retrieving the content of a chunk is time-consuming. The rationale is simply that this is an extra step that is required when there are statistical regularities than when there are not. We do *not* argue that retrieval from an offline state must always be slower than accessing information in WM, a generalization that fails in many different scenarios (e.g., reporting the capitals of known countries vs. capitals of hypothetical countries that were just learned). Nevertheless, our data are well explained by the hypothesis that subjects took longer to retrieve the content of a chunk because it required them to access associative memories.

### Memory Scanning

One may potentially suggest that, although the slowed response times in Experiments 1–4 are consistent with the hypothesis that accessing a chunk requires retrieval from offline representations, these findings could also reflect a simple increase in the time required for “memory scanning” over larger numbers of representations in WM^26^. There are a few reasons to question the validity of this alternative account.

First, our response deadline studies showed that there was little benefit in the patterned condition over control condition in brief response deadlines, suggesting that the additional regularity-based information was unavailable. One may suggest that this lack of an advantage in the patterned condition (with brief response deadlines) is due to the cost of scanning a larger set of memorized items. However, this scanning cost is based on the assumption of a “random-order scanning” process in which the spatial cue provided is ignored, and observers scan randomly until they happen to encounter a representation that matches the position of the spatial cue. This kind of random scanning seems unlikely, particularly in light of the fact that spatial position is an essential and salient part of these memory representations. By contrast, if subjects first consult the item in memory in the cued position, it is clear that a larger number of items in WM would yield better performance at the earliest response deadlines.

Second, the memory scanning account presumes that this scanning process takes hundreds of milliseconds per item, far longer than the typical scanning costs estimated from past memory scanning paradigms^26^.

Third, the conceptual necessity of the “memory scanning” hypothesis can be questioned. Clearly, in the laborious cases such as remembering the string “internationalizationcongratulationmisinterpretation”, it seems absurd to assume all 51 letters are individually hold in an online system which takes minutes to scan, and one will have to agree on the use of content-free labels. Therefore, content-free label is a necessary notion and it is theoretically more parsimonious to assume that it also accounts for the more moderate cases in the present experiments. Of course, it is possible that the RT costs in the laborious cases and those in the moderate cases are fundamentally different. However, it seems to us that the only empirical difference is that the RT costs can be easily verified by introspection in the former case but has to be experimentally measured in the latter case, and this is not a good conceptual reason to assume a theoretical dichotomy.

Fourth, and most decisively, the sequence order effect in Experiments 1–4 cannot be explained by increased scanning time in the patterned condition because the amount of stored information was identical regardless of whether top color or bottom color was tested, or whether left-side letter or right-side letter was tested. In other words, if both top-color and bottom-color of a pair are individually encoded in visual WM and the longer response time was simply due to the “scanning of more items”, then there is no reason to believe that the response should be slower for bottom-color than for top-color. On the other hand, this sequence-order can be naturally explained by assuming the content of a chunk is retrieved in a stereotyped order.

### Chunking vs. Partial Memory

Brady *et al*.^1^ considered and ruled out an alternative account in which the observers have remembered one color from each pair and use that to infer the other color. It should be clarified that the chunking account of the present study is fundamentally different from this “partial memory” account. Basically, in chunking, the observers remember a content-free label and use that label to “infer” the content of the chunk. For example, in the chunking account, the string “dogduckdolphin” is remembered as 3 animal concepts, but in the “partial memory” account, this string needs to be remembered as “ddd”, which causes confusion in this case because the “d” could be followed by different letters.

Brady *et al*.^1^ provided a few findings against the “partial memory” account. First, the memory for low-probability pairs was also better when they were stored with high probability pairs, while a strategy of guessing based on the value of one item in a pair predicts worse performance for low probability pairs. This finding is also consistent with the chunking account because, like Brady *et al*. ^1^ have assumed, the chunking of high-probability pairs could have saved representational resources for low-probability pairs even if they are not remembered as chunks. For another, Brady *et al*.^1^ found that when observers make guesses, they were not more likely to guess the high-probability partner of an item when a low probability pair was tested. For example, if red is usually paired with green but is paired with yellow in a trial, then if observers fail to report the color “yellow”, they are no more likely to report “green” than another irrelevant color such as “blue”. This finding is also very consistent with the chunking account. Although the observers will use a label to represent the regular pair “red-green”, they will not use this label in the case of “red-yellow”, and there is no reason to believe that they would be especially likely to guess “green” in that case.

### Labeling of Perceptual Information

This chunking process may also be important in perceptual input itself, in addition to the memorized information. For example, following the Boolean map theory^27^, it was proposed that the features in a familiar pattern (e.g., colors of the Star and Stripes Flag) may be consciously accessed as a whole label, and the colors themselves are not directly represented, but are *inversely inferred* from this “flag” label^28^. It has been predicted, and confirmed, that there is no familiarity-based benefit for aspects of the features that are orthogonal to the familiarity (e.g., dark red vs. light red in the Star and Stripes flag)^28^.

### Subjective Experience of the offline Representations

At the first sight, the reliance on offline representations may lead one to a rather strange picture of how they would be subjectively experienced. An observer is not faced with colored objects, but rather only “empty” regions with abstract labels attached on them. Therefore, one may find this notion strange because it is inconsistent with the intuition that real colors, not abstract labels, are kept in memory.

However, this interpretation may be misleading because the vivid subjective experience of memorizing real colors could well be built upon the mechanism of offline representations. As Dennett^29^ pointed out, human observers may often misinterpret the information that can be readily fetched as what has been represented at the moment, and regard the information as part of their subjective experience. Dennett^29^ illustrated the case of periphery vision as such an example. Human observers do not have “clear images” of the peripheral objects represented in their visual systems, yet they subjectively perceive these peripheral objects as clear rather than blurry, perhaps because the observers can obtain “clear images” of these objects by fixating on them whenever they want. Similarly, even if the online representations has only directly represented the labels, the subjective impression of “remembering the colors” may still readily emerge because the colors indicated by these labels can be retrieved from offline representations in an efficient manner.

One line of evidence consistent with this view is from the studies of change blindness^30,31^ which showed that human observers are surprisingly poor at detecting changes in their environments when they have a strong impression of seeing the whole scene at once. So, perhaps our subjectively rich and detailed visual experiences belie a persistent role for offline representations in our mental representations of the world.
